# Interpreting change from patient reported outcome (PRO) endpoints: patient global ratings of concept versus patient global ratings of change, a case study among osteoporosis patients

**DOI:** 10.1186/s12955-016-0427-5

**Published:** 2016-02-19

**Authors:** Annabel Nixon, Helen Doll, Cicely Kerr, Russel Burge, April N. Naegeli

**Affiliations:** Chilli Consultancy, Salisbury, UK; ICON Patient Reported Outcomes, Oxford, UK; Formerly ICON Patient Reported Outcomes, Oxford, UK; Eli Lilly and Company LCC, DC, 1730, 46285 Indianapolis, IN USA

**Keywords:** Patient reported outcomes, Interpretation of change, Osteoporosis, OPAQ-PF, Global ratings, Anchor-based methods

## Abstract

**Background:**

Regulatory guidance recommends anchor-based methods for interpretation of treatment effects measured by PRO endpoints. Methodological pros and cons of patient global ratings of change vs. patient global ratings of concept have been discussed but empirical evidence in support of either approach is lacking. This study evaluated the performance of patient global ratings of change and patient global ratings of concept for interpreting patient stability and patient improvement.

**Methods:**

Patient global ratings of change and patient global ratings of concept were included in a psychometric validation study of an osteoporosis-targeted PRO instrument (the OPAQ-PF) to assess its ability to detect change and to derive responder definitions. 144 female osteoporosis patients with (*n* = 37) or without (*n* = 107) a recent (within 6 weeks) fragility fracture completed the OPAQ-PF and global items at baseline, 2 weeks (no recent fracture), and 12 weeks (recent fracture) post-baseline.

**Results:**

Results differed between the two methods. Recent fracture patients reported more *improvement* while patients without recent fracture reported more *stability* on ratings of change than ratings of concept. However, correlations with OPAQ-PF score change were stronger for ratings of concept than ratings of change (both groups). Effect sizes for OPAQ-PF score change increased consistently with level of change in ratings of concept but inconsistently with ratings of change, with the mean AUC for prediction of a one-point change being 0.72 vs. 0.56.

**Conclusions:**

This study provides initial empirical support for methodological and regulatory recommendations to use patient global ratings of concept rather than ratings of change when interpreting change captured by PRO instruments in studies evaluating treatment effects. These findings warrant being confirmed in a purpose-designed larger scale analysis.

**Electronic supplementary material:**

The online version of this article (doi:10.1186/s12955-016-0427-5) contains supplementary material, which is available to authorized users.

## Background

As with all outcome measures used to evaluate the impact of a medical product, one of the biggest challenges for patient reported outcome (PRO) endpoints is how to interpret the change in scores between two time points or the difference in change scores between treatment groups [1]. Most PRO instruments comprise one or more scales, with items aggregated into multi-item scales and scores for each individual often considered at group-level. Interpretation of PRO data requires an understanding of potential complexities associated with self-report, comparison of different response scales, and of psychometrics in general [1]. Further, the statistical significance of any score change over time does not guarantee that differences are clinically meaningful, with statistical significance sometimes achieved for notably small score changes, particularly if the sample size is large [2, 3].

The history of debate over the methods for interpreting change in longitudinal studies has been recently summarised by Wyrwich and colleagues [4]. The debate heightened during the consultation period following publication of the Food and Drug Administration (FDA) draft guidance [5] and continued after the publication of the FDA final guidance report for Industry PRO measures [2], with Burke and Trentacosti [6] offering further considerations beyond those provided in the final FDA guidance. A key shift in approach over this period has been a move away from using the group level minimum important difference (MID) to evaluate treatment benefit, the focus of much of the prior efforts to develop values to aid interpretation of change [4], to using the patient level responder definition. The MID is the amount of difference between treatment groups in the change observed in a PRO measure that can be interpreted as a treatment benefit [5], whereas the responder definition is the amount of change *in an individual patient* which can be interpreted as a treatment benefit: the proportions of individuals in each trial arm who meet this threshold (or indeed a variety of thresholds) for PRO score change are compared between treatment arms.

Empirical approaches for defining both the MID and the responder definition can be either anchor-based or distribution-based. The FDA has stated a preference for anchor-based rather than distribution-based approaches for establishing the responder definition [2]. Anchor-based methods explore the association between the PRO instrument and a related external anchor, where different types of anchors can be utilised. Distribution-based approaches evaluate score change in the context of score variability e.g. ½ standard deviation, or 1 standard error of measurement (SEM). Typically, distribution-based approaches have been used to establish the MID. Where anchor-based approaches have been used, this tends to be termed minimum clinically important difference (MCID), although these terms have been used interchangeably. The FDA considers distribution-based approaches to be supportive of anchor-based approaches, providing the minimum value for a responder definition derived from anchor-based methods. This is because a responder definition must be at least large enough to be beyond a score change that might reasonably be expected by chance alone [2].

The FDA PRO Guidance [2] makes specific reference to the use of patient ratings of change as an anchor, for which the patient rates how much change they have experienced on a single-item scale. Importantly, this scale must relate conceptually to the content of the PRO instrument that will be used to evaluate treatment benefit (e.g., pain or physical function), an approach originally developed by Jaeschke et al. [7] and the most commonly reported anchor-based method. This single-item scale has response options ranging from deterioration through to improvement in the concept of interest, with a mid-point of no change. The corresponding PRO score change for patients who have rated a certain, such as a small or moderate, level of change is then taken to indicate a meaningful PRO score change for use in PRO score interpretation and to identify responders (i.e., those whose level of PRO score change has have met the responder definition). There are, however, concerns about the use of patient global ratings of change, specifically concerning recall bias associated with retrospective assessments over long periods of time [8] and for diseases with a high level of symptom variability across short periods of time, such as irritable bowel syndrome [6]. Thus, the FDA has more recently recommended the use of patient global ratings of concept items, for which the patient rates their current state on a relevant PRO concept at each key time-point, with the change in global concept ratings between the time-points calculated for analysis [6].

Whilst methodological pros and cons of patient global ratings of change versus change in patient global ratings of concept have been discussed [4], empirical evidence for the relative benefits of each approach is lacking. This study compares the statistical performance of two anchor based approaches, patient global rating of change and patient global rating of concept, for interpreting patient stability and patient improvement using data collected for the psychometric validation of an osteoporosis-targeted PRO, the Osteoporosis Assessment Questionnaire – Physical Function (OPAQ-PF).

## Methods

The design, sample and procedures of the full OPAQ-PF psychometric validation study are reported in detail elsewhere [9].

### Design

**Fig. 1:**
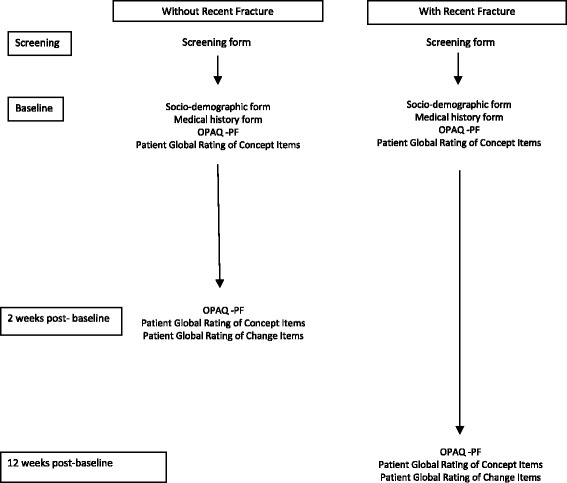
Schedule of assessments

Post-menopausal women aged ≥50 years, diagnosed with moderate-to-severe osteoporosis, with or without a recent (within six weeks prior to baseline) fragility fracture, were recruited through ten clinical sites in the US. These patients completed the OPAQ-PF and global items at baseline (global concept), 2 weeks (no recent fracture, global concept and change) and 12 weeks (recent fracture, global concept and change) post-baseline (Fig. 1). Participants who had not experienced a recent fracture were expected to experience stability in their ability to perform daily activities of physical functioning at week two while those with recent osteoporotic fracture were expected to experience change, specifically improvement, in their ability to perform daily activities of physical functioning between baseline and week 12. Institutional review board (IRB) approval was obtained for the study (Protocol OXO2550; Independent Investigational Review Board, Inc.: 21 October 2011).

### Measures

The OPAQ-PF [9, 10] is designed to evaluate the participant’s ability to perform their daily activities of physical function during the past seven days. The instrument covers mobility (5 items), physical positions (6 items) and transfers (4 items). Items are rated on a six-point Likert response scale ranging from ‘no difficulty’ [score 0] to ‘completely avoided doing this’ [score 5] (subsequently modified to ‘unable to do’; see discussion). All 15 items are reverse scored, summed and transformed to a 0–100 scale to provide a total score, where 0 indicates the most difficulties and 100 no difficulties. A qualitative study with 32 participants demonstrated content validity of the OPAQ-PF in post-menopausal women who had, and had not, previously sustained a fracture [9]. A prospective study of 144 postmenopausal women with moderate to severe osteoporosis demonstrated that the OPAQ-PF was: unidimensional; had good internal consistency (α = 0.974); good test-retest reliability (ICC = 0.993); differentiated between patients with/without a recent fracture and by severity of osteoporosis; and correlated strongly with hypothesized-related scales and performance based measures (r ≥ 0.6, *p* < 0.001) [10].

Three patient global ratings of concept items (ratings of concept) and three patient global ratings of change items (ratings of change) were developed to evaluate the ability of the OPAQ-PF to detect change and to evaluate interpretation of change [4]. These ratings reflected the three content areas of the OPAQ-PF (mobility, physical positions and transfers). Ratings of concept items were self-completed by participants to reflect overall difficulty in the last seven days in these areas due to osteoporosis. For example, for mobility participants were asked “Overall, how much difficulty have you had with mobility (e.g. walking or climbing stairs) due to your osteoporosis in the last 7 days?” The participant rated difficulty on a five-point scale ranging from ‘no difficulty’ (0), through ‘a little difficulty’ (1), ‘some difficulty’ (2), ‘moderate difficulty’ (4), to ‘severe difficulty’ (5). Ratings of change items were self-completed by participants who were asked to rate their overall change in the same three areas since the last study visit. For example, the mobility rating of change item asked “Overall, compared to your last visit, how has your mobility (e.g. ability to walk or climbing stairs) due to your osteoporosis changed?” Participants rated each item on a seven-point scale ranging from ‘much better’ (3), through ‘moderately better’ (2), ‘a little better’ (1), ‘no change’ (0), ‘a little worse’ (−1), ‘moderately worse’ (−2), to ‘much worse’ (−3).

### Procedures

Full study procedures are reported elsewhere [10]. Participants completed the OPAQ-PF and the three ratings of concept at baseline. Participants without a recent fracture completed the OPAQ-PF, the three ratings of concept, and three ratings of change two weeks (median 14 days, Inter-Quartile Range (IQR) 14–18 days) after baseline, over which time change was not expected. Participants with a recent fracture attended a visit at 12 weeks (median 12, IQR 12.0-12.7) post-baseline and completed the OPAQ-PF, the three ratings of concept and three ratings of change items at each follow-up visit (Fig. 1). Improvement was expected among recent fracture participants during this time period. Participants with a recent fracture also attended a visit at 24 weeks post-baseline [10]; these data are not used in the current analysis because the rating of change items asked the patients to compare their functioning with the previous visit (week 12) rather than baseline.

### Statistical analysis

The change on each rating of concept was calculated at week two (no recent fracture group) and week 12 (recent fracture group) relative to the score at baseline. In order to evaluate possible recall bias, ratings of change were correlated with ratings of concept completed at the same and previous time-point using Spearman’s correlation coefficient (r_s_). Retrospective recall is considered unbiased if ratings of change are positively correlated with follow-up scores and negatively correlated with baseline scores [8], and to an equal degree [11]. Correlations were expected to be at least moderate at ≥ |0.30| [12]. Change scores in OPAQ-PF at weeks two and 12 were calculated and OPAQ-PF score changes correlated with patient global ratings of concept and patient global ratings of change scores (r_s_). Mean and median OPAQ-PF change scores were compared between participants in each patient-rated level of change and change in patient ratings of concept group using ANOVA and Kruskal-Wallis (K-W) tests. Tests for linear trend (and departures from linearity) were conducted within the ANOVA. Cohen’s *d* effect size for OPAQ-PF change (mean change / baseline standard deviation, SD; [12]) were calculated at each level of rating of change and change in ratings of concept.

Receiver operator characteristic (ROC) curves were used to identify the OPAQ-PF change score which best distinguishes individual patients who improved to a specified extent from those who did not (the ‘best cut point’, BCP) [13, 14]. ROC curves were plotted for participants reporting at least a one unit improvement on the specific ratings of concept and ratings of change at weeks 2 (no recent fracture group) and 12 weeks (recent fracture group). ROC curves plot sensitivity, the proportion of true ‘positives’ detected (*y*-axis) against 1-specificity, the proportion of true ‘negatives’ detected (*x*-axis) for all possible cut-points of the OPAQ-PF. The ‘best cut point’ is identified as the test value which maximises the sum of sensitivity and specificity, i.e. the test value associated with the point closest to the top left hand corner of the ROC space. The area under the curve (AUC) can also be calculated: the closer it is to 1.0, the better the differentiation of the scale. Therefore, the greater the AUC, the greater the ability of the OPAQ-PF to differentiate those who reported change from those who did not.

Statistical significance throughout was taken at the 5 % level (*p* < 0.05).

## Results

### Sample

The overall sample (*n* = 144), comprised 107 patients without recent fracture and 37 recent fracture patients. Baseline sample characteristics are presented in Table 1 (further details reported elsewhere [10]).

**Table 1:** Participant characteristics

N (%)	No recent fracture (*N* = 107)	Recent fracture (*N* = 37)	Total (*N* = 144)
Age, years, mean (SD) [min-max, years]	69.7 (8.70) [52.0-88.8]	69.4 (9.70) [51.3-87.9]	69.4 (9.70) [51.3-88.8]
Education			
Did not complete high school	5 (4.7 %)	3 (8.1 %)	8 (5.6 %)
High school	31 (29.0 %)	12 (32.4 %)	43 (29.9 %)
Some college	33 (30.8 %)	12 (32.4 %)	45 (31.2 %)
College degree	25 (23.4 %)	4 (10.8 %)	29 (20.1 %)
Graduate degree	13 (12.1 %)	6 (16.2 %)	19 (13.2 %)
Ethnicity			
Asian	2 (1.9 %)	0 (0 %)	2 (1.4 %)
Black	1 (0.9 %)	3 (8.1 %)	4 (2.8 %)
Hispanic	4 (3.7 %)	2 (5.4 %)	6 (4.2 %)
White or Caucasian	100 (93.5 %)	32 (86.5 %)	132 (91.7 %)
Employment			
Employed full time	14 (13.1 %)	6 (16.2 %)	20 (13.9 %)
Employed part time	8 (7.5 %)	0 (0 %)	8 (5.6 %)
Retired	64 (59.8 %)	21 (56.8 %)	85 (59.0 %)
Unemployed	1 (0.9 %)	0 (0 %)	1 (0.7 %)
Looking after home/family	11 (10.3 %)	2 (5.4 %)	13 (9.0 %)
Permanently unable to work	7 (6.5 %)	7 (18.9 %)	14 (9.7 %)
Other	2 (1.9 %)	1 (2.7 %)	3 (2.1 %)
Disease duration [min-max, years]	6.83 (4.80) *n* = 94 [0.45 – 19.8]	8.02 (6.75) *n* = 25 [0–30.6]	7.08 (5.26) *n* = 119 [0 – 30.6]
Time (months) since most recent fracture [min-max, months]	123.9 (193.5) *n* = 53 [1.97 - 703.8]	0.75 (0.41) *n* = 37 [0–1.35]	73.3 (160.0) *n* = 90 [0–704.6]
Medication			
Calcium	69 (64.5 %)	24 (64.9 %)	93 (64.6 %)
Vitamin D	73 (68.2 %)	24 (64.9 %)	97 (67.4 %)
Bisphosphonates	68 (63.6 %)	12 (32.4 %)	80 (55.6 %)
Other	29 (27.1 %)	9 (24.3 %)	38 (26.4 %)
Pain medications^a^	64 (59.8 %)	28 (75.7 %)	92 (63.9 %)
Upper/lower limb impediments			
Osteoarthritis	42 (39.3 %)	14 (37.8 %)	56 (38.9 %)
Hip	22 (20.6 %)	3 (8.1 %)	25 (17.4 %)
Knee	32 (29.9 %)	10 (27.0 %)	42 (29.2 %)
Ankle	5 (4.7 %)	3 (8.1 %)	8 (5.6 %)
Shoulder	11 (10.3 %)	3 (8.1 %)	14 (9.7 %)
Wrist/hand	25 (23.4 %)	9 (24.3 %)	34 (23.6 %)
Other upper limb	12 (11.2 %)	3 (8.1 %)	15 (10.4 %)
Other lower limb	14 (13.1 %)	6 (16.2 %)	20 (13.9 %)
No upper/lower limb impediment	57 (53.3 %)	20 (54.1 %)	77 (53.5 %)
OPAQ-PF scores (Total 0–100)^b^			
Mean (SD)	82.2 (21.1)	57.0 (26.1)	75.7 (24.9)
Median (IQR)	92.0 (69.3–100)	58.7 (37.3–78.7)	83.3 (57.7–98.7)
Min, max	28, 100	5.33, 100	5.33, 100
N (%) max	31 (29.0 %)	1 (2.7 %)	32 (22.2 %)

^a^includes 8 patients who reported taking pain medications their clinicians were unaware of and 5 patients who did not report taking medications prescribed by their clinicians

^b^lower scores indicate greater impairment

### Patient ratings of change and change in global concept

**Fig. 2:**
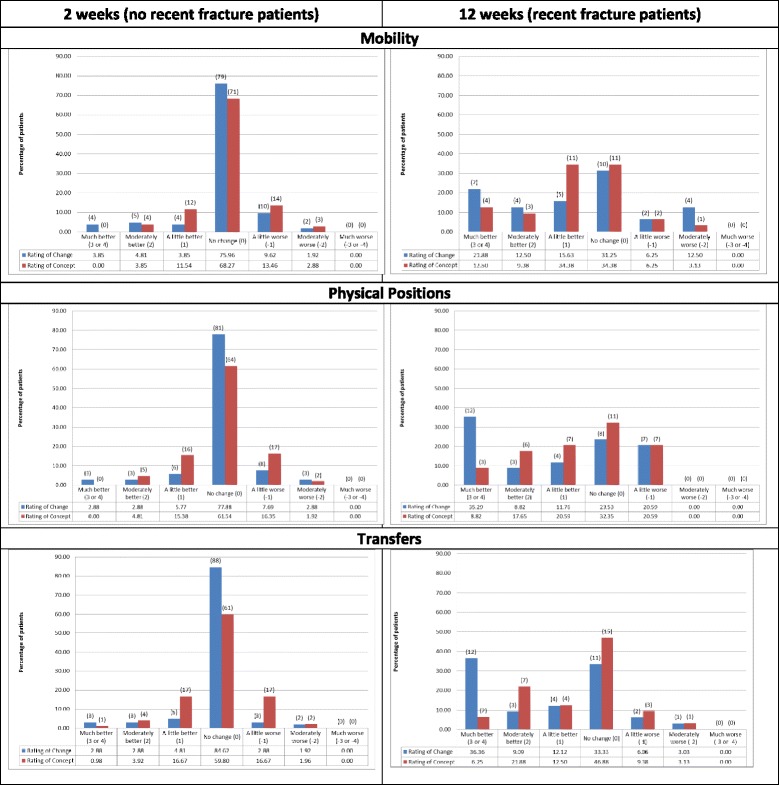
Patient % reporting each mobility, physical positions, and transfers global change/difference in concept rating at each assessment. Note: Numbers in brackets are the number of subjects in each response category

At week 2, the no recent fracture patients were more likely to report stability (no change) on the ratings of change than on the ratings of concept (i.e., difference in ratings of concept between time-points = 0): mobility *n* = 79 (75 %) vs. *n* = 72 (69 %), physical positions *n* = 81 (77 %) vs. *n* = 65 (62 %), transfers *n* = 88 (84 %) vs. *n* = 62 (60 %) (Fig. 2). At week 12, the recent fracture patients were generally less likely to report stability or a small degree of improvement on the ratings of change, being instead much more likely to report feeling’much better’ vs. an improvement of 3 or more on the ratings of concept: mobility *n* = 12 (35 %) vs. *n* = 2 (6 %), physical positions *n* = 12 (35 %) vs. *n* = 3 (9 %), transfers *n* = 12 (36 %) vs. *n* = 2 (6 %) (Fig. 2).

Patient ratings of change at 2 and 12 weeks were at least moderate and correlated significantly with patient ratings of concept at the same assessment, but were smaller and (at 12 weeks) generally not significantly correlated with ratings of concept at the previous assessment. Thus, the correlations between 2-week ratings of change and concept were: mobility 0.322 (*p* < 0.001), physical positions 0.328 (*p* < 0.001), transfers 0.300 (*p* = 0.002); whereas correlations between 2-week ratings of change and baseline ratings of concept were: mobility 0.216 (*p* = 0.027), physical positions 0.258 (*p* = 0.008), transfers 0.200 (*p* = 0.041). Correlations between 12-week ratings of change and concept were: mobility 0.699 (*p* < 0.001), physical positions 0.694 (*p* < 0.001), and transfers 0.481 (*p* = 0.005); whereas correlations between 12-week ratings of change and baseline ratings of concept were 0.451 (*p* = 0.007), 0.271 (*p* = 0.121), and −0.037 (*p* = 0.843) respectively.

### Correlation with OPAQ-PF score change

Correlations with the OPAQ-PF score change were stronger for change in ratings of concept (Table 2) than ratings of change (Table 3). At week 2, correlations for the change in the ratings of concept items were 0.152 (*p* = 0.122) for Mobility, 0.247 (*p* = 0.011) for Physical Positions and 0.318 (*p* < 0.01) for Transfers, compared with −0.082 (*p* = 0.405) for Mobility, 0.042 (*p* = 0.673) for Physical Positions, and 0.028 (*p* = 0.777) for Transfers ratings of change. At week 12, correlations for the change in ratings of concept items were 0.749 (*p* < 0.01) for Mobility, 0.464 (*p* < 0.01) for Physical Positions and 0.503 (*p* < 0.01) for Transfers compared with 0.322 (*p* = 0.064) for Mobility, 0.447 (*p* < 0.01) for Physical Positions, and 0.426 (*p* = 0.013) for Transfers ratings of change.

**Table 2:** Mean OPAQ-PF total scores at baseline, change scores, and effect sizes at weeks 2 (no recent fracture patients) and 12 (recent fracture patients) by changes in patient global ratings of concept at weeks 2 and 12

	No recent fracture patients (*N* = 105)				Recent fracture patients (*N* = 34)			
Change in rating of concept		Baseline	2 weeks	Effect size^a^ for OPAQ-PF change		Baseline	12 weeks	Effect size^a^ for OPAQ-PF change
	N	OPAQ-PF total score mean (SD)	OPAQ-PF mean change (SD)		N	OPAQ-PF total score mean (SD)	OPAQ-PF mean change (SD)	
Mobility								
3 or 4	0	-	-	-	2	63.3 (51.9)	22.7 (71.7)	0.44
2	4	80.0 (26.8)	2.33 (24.2)	0.09	4	42.3 (6.00)	37.7 (16.1)	6.28
1	12	84.6 (14.5)	2.33 (12.0)	0.16	12	55.9 (20.6)	19.0 (18.0)	0.92
0	72	81.1 (22.6)	−2.54 (8.89)	−0.11	15	64.4 (28.6)	3.91 (21.7)	0.14
−1	14	83.9 (22.3)	−3.33 (8.57)	−0.15	1	69.3	−9.33	-
−2	3	69.6 (30.3)	−6.49 (13.1)	−0.21	0	-	-	-
−3 or −4	0	-	-	-	0	-	-	-
	K-W test *p*-value		0.475				0.069	
	Linear trend *p*-value (departures from linear trend)		0.079 (0.848)				0.015 (0.606)	
	r_s_ (*p*-value)		0.152 (0.122)				0.464 (0.006)	
Physical Positions								
3 or 4	0	-	-	-	3	38.2 (10.1)	50.2 (27.4)	4.97
2	5	61.3 (35.1)	4.80 (17.6)	0.14	6	44.4 (10.3)	35.8 (13.4)	3.48
1	16	83.5 (17.2)	0.00 (5.74)	0.0	7	61.7 (17.4)	14.3 (18.5)	0.82
0	65	82.4 (21.8)	−1.83 (10.2)	0.08	11	59.2 (34.4)	3.39 (22.1)	0.10
−1	17	81.0 (21.2)	−6.40 (9.80)	−0.31	7	77.0 (15.7)	−4.19 (13.9)	−0.27
−2	2	94.0 (6.60)	−4.00 (7.54)	−0.61	0	-	-	-
−3 or −4	0	-	-	-	0	-	-	-
	K-W test *p*-value		0.150				<0.005	
	Linear trend *p*-value (departures from linear trend)		0.021 (0.840)				<0.001 (0.763)	
	r_s_ (*p*-value)		0.247 (0.011)				0.749 (<.001)	
Transfers^b^								
3 or 4	1	73.3 (−)	6.67 (−)	-	2	44.0 (1.89)	38.7 (26.4)	20.5
2	4	64.7 (23.4)	1.33 (16.7)	0.12	7	45.2 (15.3)	37.0 (22.5)	2.42
1	17	79.1 (15.5)	0.78 (6.00)	0.05	4	65.7 (31.2)	13.3 (28.9)	0.43
0	62	84.8 (22.4)	−1.96 (10.3)	−0.09	15	64.4 (29.9)	2.58 (20.5)	0.09
−1	17	78.2 (24.4)	−4.91 (9.99)	−0.20	3	64.0 (13.1)	14.2 (16.9)	0.83
−2	2	76.7 (2.83)	−17.3 (5.66)	−6.11	1	40.0 (−)	10.7 (−)	-
−3 or −4	0	-	-	-	0	-	-	-
	K-W test		0.015				0.063	
	Linear trend *p*-value (departures from linear trend)		0.010 (0.742)				0.005 (0.332)	
	r_s_ (*p*-value)		0.318 (0.001)				0.503 (0.003)	

Data are shown for patients with data at each two assessments

^a^Cohen’s d effect size = mean change / baseline SD

^b^ *N* = 103 (no recent fracture) *N* = 32 (recent fracture)

**Table 3:** Mean OPAQ-PF total scores at baseline, change scores, and effect sizes at weeks 2 (no recent fracture patients and 12 (recent fracture patients) by patient global ratings of change at weeks 2 and 12

	No recent fracture patients (*N* = 105)				Recent fracture patients (*N* = 34)			
Rating of change		Baseline	2 weeks	Effect size^a^ for OPAQ-PF change		Baseline	12 weeks	Effect size^a^ for OPAQ-PF change
	N	OPAQ-PF total score mean (SD)	OPAQ-PF mean change (SD)		N	OPAQ-PF total score mean (SD)	OPAQ-PF mean change (SD)	
Mobility								
Much better	4	91.3 (7.96)	−0.67 (4.93)	−0.08	12	70.4 (25.8)	19.9 (27.9)	0.77
Moderately better	5	89.6 (11.2)	−4.53 (5.47)	−0.40	6	42.7 (20.4)	29.1 (19.4)	1.43
A little better	4	89.7 (9.70)	−8.00 (13.5)	−0.82	3	61.3 (26.0)	−1.78 (47.2)	−0.07
No change	79	82.5 (22.1)	−0.38 (9.23)	−0.02	8	51.3 (27.5)	6.83 (10.7)	0.25
A little worse	10	77.3 (14.0)	−10.7 (14.4)	−0.76	3	64.9 (21.6)	−4.89 (16.0)	−0.23
Moderately worse	2	38.7 (17.0)	−1.33 (11.3)	−0.08	2	56.0 (3.8)	12.7 (23.6)	3.34
Much worse	1	22.7 (−)	−14.7 (−)	-	-	-	-	-
	K-W test *p*-value		0.148				0.24	
	Linear trend *p*-value (departures from linear trend)		0.33 (0.033)				0.086 (0.49)	
	r_s_ (*p*-value)		−0.082 (0.405)				0.322 (0.064)	
Physical Positions								
Much better	3	92.0 (9.61)	1.33 (3.53)	0.14	12	70.6 (25.6)	21.9 (28.7)	0.86
Moderately better	3	89.8 (14.4)	−5.78 (6.84)	−0.40	3	38.2 (6.30)	38.7 (16.2)	6.14
A little better	6	91.6 (6.29)	−8.44 (8.95)	−1.34	4	39.3 (17.7)	19.7 (11.4)	1.11
No change	81	82.7 (21.6)	−0.87 (9.58)	−0.04	8	52.5 (28.5)	8.67 (8.58)	0.30
A little worse	8	77.3 (12.6)	−7.83 (16.0)	−0.62	7	66.3 (16.6)	−7.62 (26.2)	−0.46
Moderately worse	3	40.9 (12.6)	0 (8.33)	0.0	-	-	-	-
Much worse	1	22.7 (−)	−14.7 (−)	-	-	-	-	-
	K-W test *p*-value		0.224				0.033	
	Linear trend *p*-value (departures from linear trend)		0.631 (0.131)				0.007 (0.310)	
	r_s_ (*p*-value)		0.042 (0.673)				0.447 (0.008)	
Transfers^b^								
Much better	3	89.3 (14.1)	−2.67 (9.61)	−0.19	12	65.1 (26.3)	24.6 (29.2)	0.94
Moderately better	3	90.7 (8.74)	−3.56 (3.36)	−0.41	3	42.2 (5.39)	33.8 (7.81)	6.27
A little better	5	94.7 (4.62)	−8.80 (9.96)	−1.90	4	50.3 (30.6)	1.33 (39.0)	0.04
No change	88	82.2 (21.3)	−0.87 (9.31)	−0.04	11	61.9 (26.9)	6.30 (14.4)	0.10
A little worse	3	70.7 (7.42)	−18.2 (23.3)	−2.45	2	59.3 (27.3)	3.33 (10.4)	0.12
Moderately worse	2	38.7 (17.0)	−1.33 (11.3)	−0.08	1	58.7	−4.0	-
Much worse	1	22.7 (−)	14.7 (−)	-	-	-	-	-
	K-W test *p*-value		0.304				0.133	
	Linear trend *p*-value (departures from linear trend)		0.655 (0.026)				0.030 (0.740)	
	r_s_ (*p*-value)		0.028 (0.777)				0.426 (0.013)	

Data are shown for patients with data at each two assessments

^a^Cohen’s d effect size = mean change / baseline SD

^b^ *N* = 33 (recent fracture)

### OPAQ-PF score changes by patient ratings of change and change in global concept

In terms of comparisons of OPAQ-PF change scores between the categories of ratings of concept (Table 2) and ratings of change (Table 3), while there were significant differences in scores for two of the six evaluations of global ratings of concept (Transfers at week 2, *p* < 0.05, and Physical positions at week 12, *p* < 0.01) and global ratings of change (Mobility at week 2 and Physical Positions at week 12, both *p* < 0.05), the associations were more likely to show significant linearity for the ratings of concept: Physical Positions at week 2, Transfers at week 2, Mobility at week 12 (all *p* < 0.05), Physical Positions at week 12, Transfers at week 12 (both *p* < 0.01); compared with the ratings of change: Transfers at week 12 (*p* < 0.05) and Physical Positions at week 12 (*p* < 0.01).

### Effect sizes

In line with the patterns shown for linearity, effect sizes for change in OPAQ-PF score at each time point were notably irregular across categories of ratings of change (Table 3) while generally increasing consistently by level of change for ratings of concept (Table 2 (and Additional file 1: Figure S1)). For example, at week 12, effect sizes for OPAQ-PF score change increased from 0.10 in those reporting no change in the Physical Positions concept, to 0.82 in those reporting a 1-point change, 3.48 in those reporting a 2-point change, and 4.97 in those reporting a 3 to 4-point change. In terms of Physical Positions ratings of change, OPAQ-PF effect sizes were 0.30 ‘no change’, 1.11 ‘a little better’, 6.4 ‘moderately better’, and 0.86 ‘much better’.

### ROC curves

ROC curves were obtained for the OPAQ-PF based on the ratings of concept and ratings of change for minimum of a one point change (Additional file 2: Figure S2). The characteristics of the ROC curves are summarised in Table 4 showing the AUC (with 95 % confidence intervals) and best OPAQ-PF cut-points for at least a one point improvement on the ratings of concept and rating of change items at weeks 2 and 12. The OPAQ-PF showed good ability to differentiate patients who had/had not shown a one point improvement on the ratings of concept/ratings of change, although disparities were found between the two methods with the ratings of concept generally being associated with greater predictive power of the OPAQ-PF. The ratings of change results at each time point had an overall mean AUC of 0.56 (range 0.37-0.78), with the AUC being less than 0.5 for each of the week 2 ROC curves, showing it is not predictive. For the ratings of concept, the mean AUC was 0.73 (range 0.60-0.87), and all were ≥0.5 and therefore predictive. The ratings of concept had slightly worse sensitivity but better specificity compared with the ratings of change (mean sensitivity over all time points 0.68, range 0.48-0.88 vs. mean 0.76, range 0.71-0.79; specificity mean 0.66, range 0.23-0.79 vs. mean 0.49, range 0.27-0.73), with the greater sensitivity of the ratings of change being obtained at the expense of low specificity.

**Table 4:** ROC statistics for a 1-point change in rating of concept and rating of change at weeks 2 (no recent fracture patients) and 12 (recent fracture patients): Area Under the Curve (AUC), Best Cut Point (BCP) with values of sensitivity (Se) and specificity (Sp) of OPAQ-PF change scores

	No recent fracture patients			Recent fracture patients		
	Week 2			Week 12		
	N (%)	AUC (95 % CI)	BCP (Se, Sp)	N (%)	AUC (95 % CI)	BCP (Se, Sp)
Ratings of change						
Mobility	13 (12)	0.41 (0.26-0.57) *p* = 0.31	−6.67 (0.77, 0.27)	21 (62)	0.71 (0.54, 0.89) *p* = 0.038	10.67 (0.71, 0.69)
Physical positions	12 (11)	0.37 (0.22-0.52) *p* = 0.14	−6.67 (0.75, 0.27)	19 (56)	0.78 (0.62-0.94) *p* < 0.001	10.67 (0.79, 0.73)
Transfers	11 (11)	0.37 (0.21-0.53) *p* = 0.16	−6.67 (0.73, 0.27)	19 (56)	0.74 (0.56-0.91) *p* = 0.022	10.67 (0.79,0.71)
Mean AUC/BCP Mean (Se, Sp)		0.38	−6.67 (0.75, 0.27)		0.74	10.67 (0.76, 0.71)
Overall mean AUC Mean (Se, Sp)		0.56 (range = 0.37 to 0.78) (0.76, 0.49)				
Ratings of concept						
Mobility	16(15)	0.60 (0.43-0.78) *p* = 0.19	2.0 (0.50, 0.79)	18 (53)	0.74 (0.57-0.91) *p* = 0.016	10.67 (0.78, 0.69)
Physical positions	21(20)	0.63 (0.49-0.77) *p* = 0.075	1.33 (0.48, 0.23)	16 (47)	0.87 (0.75-1.00) *p* < 0.001	12.0 (0.88, 0.78)
Transfers	22 (21)	0.68 (0.55-0.82) *p* = 0.008	0.67 (0.59, 0.77)	13 (41)	0.82 (0.66-0.98) *p* = 0.002	12.67 (0.85, 0.68)
Mean AUC/BCP >Mean (Se, Sp)		0.64	1.33 (0.52, 0.60)		0.81	11.78 (0.84, 0.72)
Overall mean AUC Mean (Se, Sp)		0.73 (range = 0.60 to 0.87) (0.68, 0.66)				

## Discussion

This study provides empirical data to support previous discussions of the methodological advantages and disadvantages of patient global ratings of change and patient global ratings of concept for interpreting PRO score change [4]. This study included osteoporosis patients who were expected to remain stable in terms of the concept of interest over a two-week period from study baseline (no recent fracture patients) and those who were expected to report improvement on the concept of interest over 12 weeks (recent fracture patients). Therefore the study design allowed for an evaluation of the performance of patient global ratings of change and patient global ratings of concept for interpreting both patient stability and improvement in the PRO of interest, the OPAQ-PF.

Substantial disparities were found between the performance of the ratings of change and ratings of concept in terms of level of change identified, but the ratings of concept consistently outperformed the ratings of change in terms of better informing interpretation of change in OPAQ-PF scores. Thus, while the patients without recent fracture were more likely to report stability (no change) two weeks after baseline on the ratings of change items, any changes which were reflected in the change in OPAQ-PF scores were more likely to be identified by the ratings of concept: correlations with OPAQ-PF score change at two weeks were higher for ratings of concept than ratings of change. Similarly, although the recent fracture patients were more likely to report substantial improvement on the ratings of change, in line with expected change, correlations with OPAQ-PF score change were stronger and more likely to be statistically significant for changes in the ratings of concept than the ratings of change. Thus, OPAQ-PF change scores were more likely to be different between the ratings of concept change than the ratings of change categories across both time points: effect sizes for OPAQ-PF score change generally increased linearly by level of ratings of concept change but showed an irregular pattern for ratings of change. The ROC curves also indicated that in terms of relative balance between sensitivity and specificity and the overall AUC, the OPAQ-PF had stronger discriminating properties in terms of the ratings of concept than those based on the ratings of change. Results are stronger for the week 12 data because of the greater likelihood of stability at week 2 rather than change.

It is important to note that as the patients completed both measures of change and concept on the same occasions the discrepancies identified in this analysis reflect differences in the way in which patients completed the ratings of change compared with the ratings of concept. At week 12, patients were required to think back 12 weeks to their baseline visit in order to evaluate change in the concept of interest (e.g. mobility). It is likely to have been a challenge for participants to think back accurately over this time period in order to be able to rate their change. Correlation analysis conducted in this study indicates a systematic bias in patient ratings of change. The greater correlation between ratings of change and ratings of concept at the same time-point, compared with correlations with baseline ratings of concept suggest patients are influenced more by how they feel currently than by an accurate assessment of how they felt previously. This is consistent with previous reports of retrospective recall of change at follow-up being positively correlated with concurrent PRO scores and either un-correlated or positively correlated with baseline scores [8, 15]. These indicate that respondents with good health at follow-up are more likely to assume that their health has recently improved, and respondents with poor health are more likely to assume that it has worsened [16].

There are issues associated with osteoporosis which are likely to increase error measurement in the patient’s self-reports. Specifically, the length of recall required in order to capture change associated with the healing of a fracture meant that patients were asked to recall over a substantial period of time (12 weeks) for the global rating of change item. Given the older age of osteoporosis patients, this length of recall may be a specific challenge for these patients leading to greater recall inaccuracies than may be experienced in other indications. It was for this reason that the global ratings of change at 24-weeks asked about change since the last visit rather than from baseline. The significant comorbidity experienced in osteoporosis may also influence the reporting on both the ratings of concept and ratings of change, as patients may find it hard to separate physical function impacts that are a specific consequence of osteoporosis from those associated with comorbidities. Specifically, over a third of the patients with a recent fracture had osteoarthritis. The global items asked patients to report their difficulty with or change in mobility, physical positions and transfers ‘due to your osteoporosis’ and the extent to which patients were able to attribute their experience to their osteoporosis or osteoarthritis was not evaluated in this analysis. It is possible that change in difficulty with mobility, physical positions and transfers may have occurred due to the patient’s osteoarthritis, which might have presented a reporting challenge to these patients or meant that these patients were not as stable as the analysis has understood them to be.

This study had several limitations, most notably that only those in the relatively small ‘recent fracture’ group in this study were hypothesized to change, and therefore there were few subjects in each of the relevant categories of change. This leads to instability of and uncertainty around the estimates calculated. This study was not purpose-designed to evaluate the research question presented, and instead represents secondary analysis of data that was designed to evaluate the psychometric measurement properties of the OPAQ-PF reported elsewhere [10]. This study is therefore limited to providing an indication of the relative performance of the two approaches. The findings from this study need to be confirmed in a purpose-designed larger-scale analysis before firm conclusions can be drawn regarding the statistical performance of patient global ratings of concept compared with patient global ratings of change.

Further limitations to the study include the fact that the sample was more suitable for evaluating stability or change in terms of improvement rather than decline. Although the ratings of change and ratings of concept allowed for report of decrement, the validation study inclusion criteria were designed to identify patients who were anticipated to remain stable (with no recent fracture history) or improve (following a recent fracture). Further work is required in order to determine whether the benefit of ratings of concept over ratings of change reported here are maintained in a study which sees patients experiencing decrement in the concept of interest. Secondly, no overall physical function global ratings of concept and change items were developed to match conceptually the final uni-dimensional structure of the OPAQ-PF; instead three patient global ratings of concept and equivalent ratings of change were developed to reflect the three content areas of the OPAQ-PF (mobility, physical positions and transfers). However, this did provide more granular results than would be possible with a single global item. Following completion of the data collection on which this analysis is based, the OPAQ-PF response option ‘completely avoided doing this’ was subsequently changed to ‘unable to do’ in line with feedback from the regulatory authorities. Finally, the impact of response shift has not been considered, where an individual’s criteria for the construct of interest changes during the course of illness and treatment, possibly leading to a modification of their internal standards, values and conceptualization of the target construct [17]. Response shift is an issue for patients reporting on ratings of concept as much as it is for those reporting on ratings of change, adding unmeasured variability not considered in our results.

## Conclusion

This study provides initial empirical support for methodological and regulatory recommendations to use patient global ratings of concept when evaluating interpretation of change for PRO instruments in studies evaluating treatment effects. It provides further evidence for the role of present state bias in leading patients systematically to overestimate their degree of improvement (or worsening) when using patient global ratings of change. These findings warrant being confirmed in a purpose-designed larger scale study.

## Additional files

Additional file 1: Figure S1. Effect sizes for OPAQ-PF total score change from baseline at 2 weeks (no recent fracture patients) and 12 weeks (recent fracture patients) by Mobility, Physical Positions, and Transfers global ratings of change and change in ratings of concept. (DOCX 169 kb) Additional file 2: Figure S2. ROC curves identifying the best cut point (BCP, indicated by the arrow) of OPAQ-PF change scores for an improvement of 1 point on Mobility, Physical Positions, and Transfers ratings of change and ratings of concept at weeks 2 (no recent fracture patients) and 12 (recent fracture patients). (DOCX 188 kb)

## References

[1] King M (2011). A point of minimal important difference (MID): a critique of terminology and methods. Expert Rev Pharmacoeconom Outcomes Res.

[2] Food and Drugs Administration (FDA). Guidance for Industry Patient-Reported Outcome Measures: Use in Medical Product Development to Support Labeling Claims. December 2009. Available from: http://www.fda.gov/downloads/Drugs/GuidanceComplianceRegulatoryInformation/Guidances/UCM193282.pdf 10.1186/1477-7525-4-79 PMC1629006 17034633

[3] Doll H, Carney S (2005). Statistical approaches to uncertainty: *p* values and confidence intervals unpacked. Evid Based Med.

[4] Wyrwich KW, Norquist JM, Lenderking WR, Acaster S, the Industry Advisory Committee of International Society for Quality of Life Research (ISOQOL) (2013). Methods for interpreting change over time in patient-reported outcome measures. Qual Life Res.

[5] Food and Drugs Administration (FDA). Guidance for Industry Patient-Reported Outcome Measures: Use in Medical Product Development to Support Labeling Claims: draft guidance. Health Qual Life Outcomes 2009; doi:10.1186/1477-7525-4-79 10.1186/1477-7525-4-79 PMC1629006 17034633

[6] Burke LB, Trentacosti AM. Interpretation of PRO trial results to support FDA labelling claims: the regulator perspective. W12, International Society for Pharmacoeconomics and Outcomes Research 15^th^ Annual International Meeting. Atlanta: GA 2010. http://www.ispor.org/meetings/atlanta0510/workshops.asp accessed 27 Apr 2015

[7] Jaeschke R, Singer J, Guyatt GH (1989). Measurement of health status. Ascertaining the minimal clinically important difference. Control Clin Trials.

[8] Norman GR, Stratford P, Regehr G (1997). Methodological problems in the retrospective computation of responsiveness to change: the lesson of Cronbach. J Clin Epidemiol.

[9] Naegeli A, Nixon A, Burge R, Gold DT, Silverman S (2014). Development of the Osteoporosis Assessment Questionnaire-Physical Function (OPAQ-PF): an osteoporosis-specific, patient-reported outcomes (PRO) measure of physical function. Osteoporos Int.

[10] Nixon A, Kerr C, Doll H, Naegeli AN, Shingler SL, Breheny K (2014). Osteoporosis Assessment Questionnaire-Physical Function (OPAQ-PF): a psychometrically validated osteoporosis-targeted patient reported outcome measure of daily activities of physical function. Osteoporosis Int.

[11] Guyatt GH, Norman GR, Juniper EF, Griffith LE (2002). A critical look at transition ratings. J Clin Epidemiol.

[12] Cohen J (1988). Statistical Power Analysis for the Behavioral Sciences.

[13] Dawson J, Doll H, Coffey J, Jenkinson C (2007). Responsiveness and minimally important change for the Manchester-Oxford foot questionnaire (MOXFQ) compared with AOFAS and SF-36 assessments following surgery for hallux valgus. Osteoarthritis Cartilage.

[14] Dawson J, Doll H, Boller I, Fitzpatrick R, Little C, Rees J (2008). Comparative responsiveness and minimal change for the Oxford Elbow Score following surgery. Qual Life Res.

[15] Cella D, Hahn E, Dineen K (2002). Meaningful change in cancer-specific quality of life scores: Differences between improvement and worsening. Qual Life Res.

[16] Knox S, King M. Validation and calibration of the SF-36 health transition question in the Household, Income and Labour Dynamics in Australia (HILDA) survey. CHERE Working Paper 2007/15. Centre for Health Economics Research and Evaluation, 2007. http://pandora.nla.gov.au/pan/81021/20080130-0950/wp2007_15.pdf.

[17] Swartz RJ, Schwartz C, Basch E, Cai L, Fairclough DL, McLeod L (2011). The king’s foot of patient-reported outcomes: current practices and new development for the measurement of change. Qual Life Res.

