# Supplementary material for: Interpreting change from patient reported outcome (PRO) endpoints: patient global ratings of concept versus patient global ratings of change, a case study among osteoporosis patients
Source: Health Qual Life Outcomes. 2016 Feb 19;14:25. doi: 10.1186/s12955-016-0427-5 (PMC4759933; doi:10.1186/s12955-016-0427-5)
Supplement: Additional file 1: Figure S1. — Effect sizes for OPAQ-PF total score change from baseline at 2 weeks (no recent fracture patients) and 12 weeks (recent fracture patients) by Mobility, Physical Positions, and Transfers global ratings of change and change in ratings of concept. (DOCX 169 kb) [file 12955_2016_427_MOESM1_ESM.docx]

*Additional file 1: Figure S1: Effect sizes for OPAQ-PF total score change from baseline at 2 weeks (no recent fracture patients) and 12 weeks (recent fracture patients) by Mobility, Physical Positions, and Transfers global ratings of change and change in ratings of concept*

| *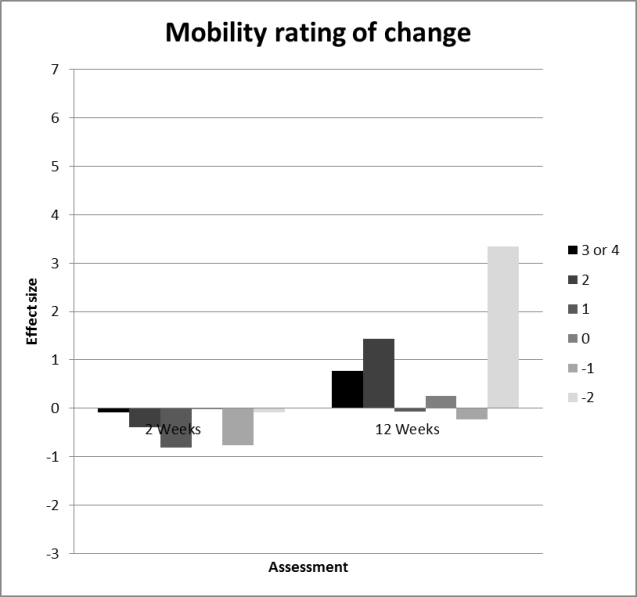* | *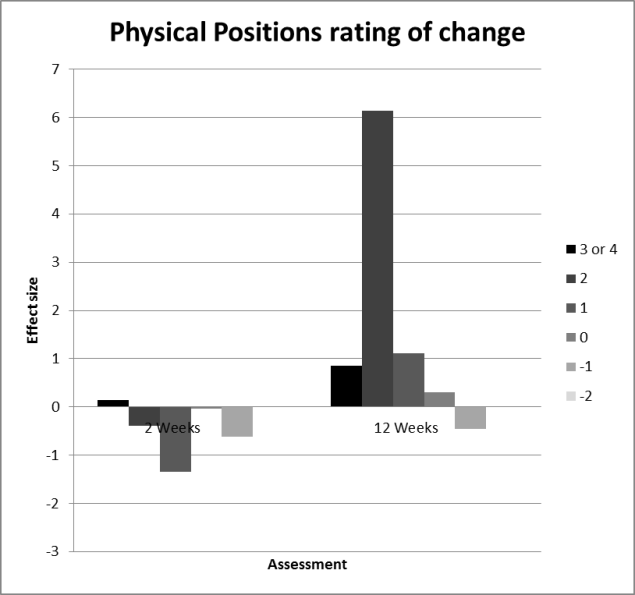* | *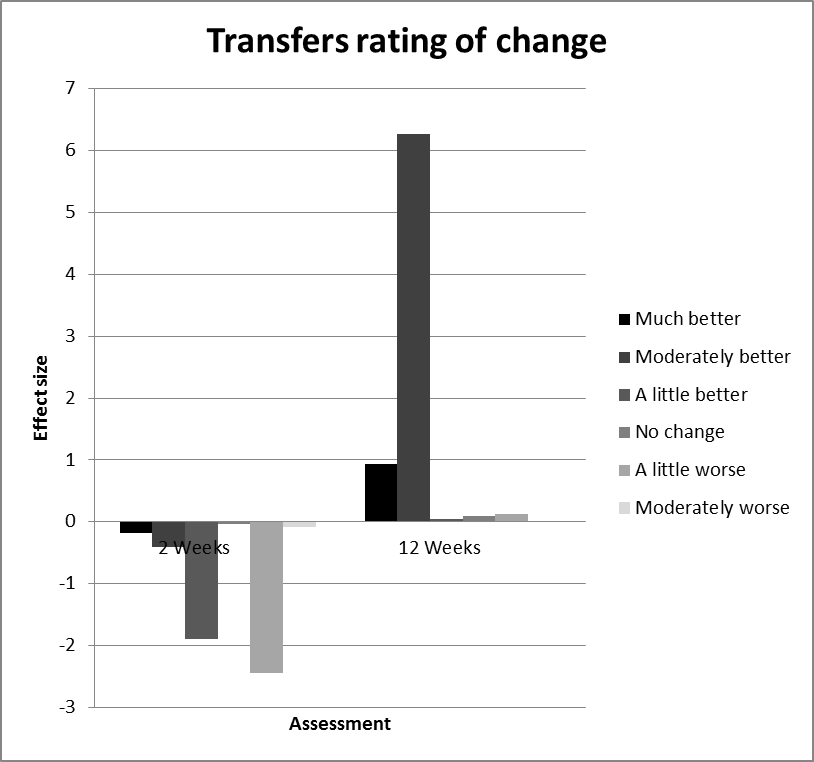* |
| --- | --- | --- |
| *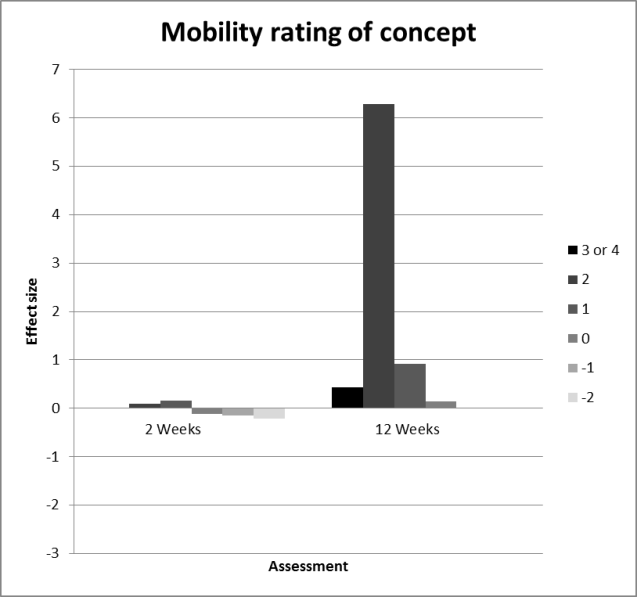* | *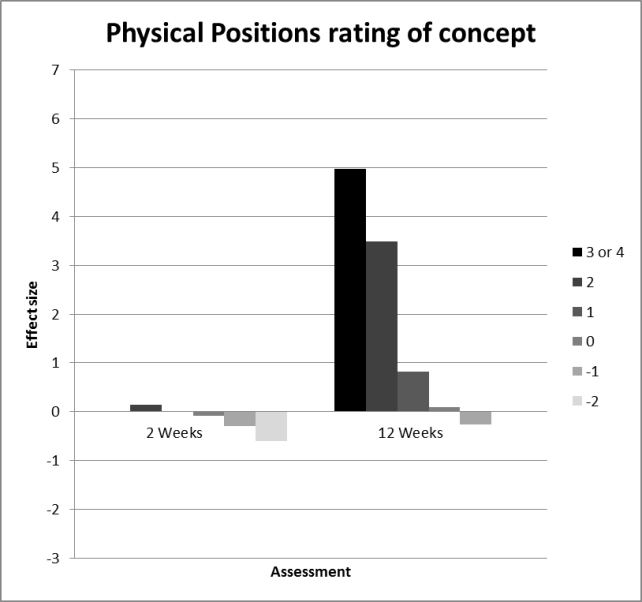* | *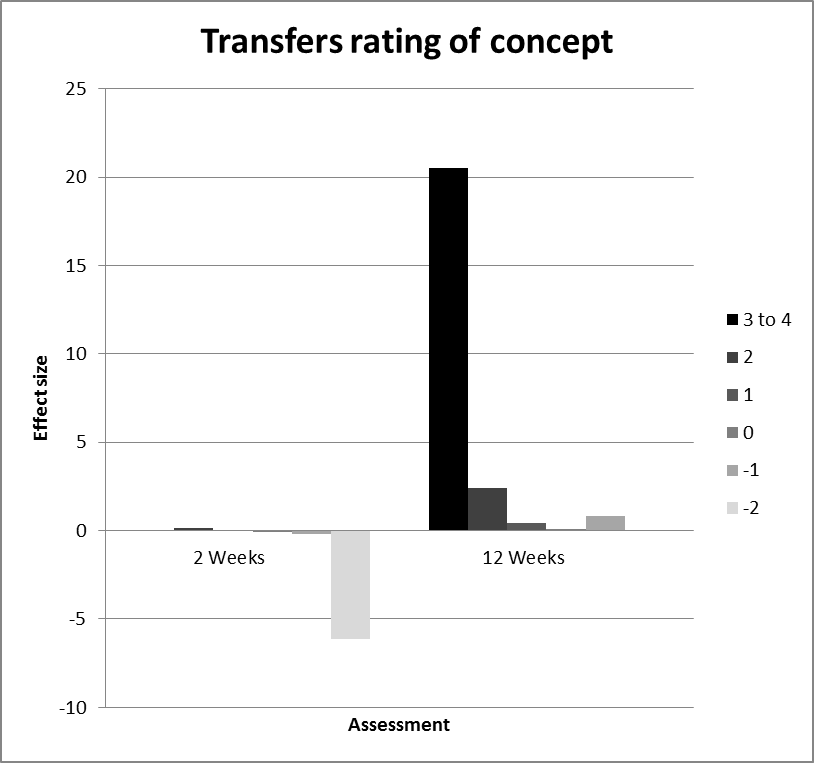* |

*Note: Transfers change in rating of concept is presented on a wider scale*
