# Supplementary material for: Interpreting change from patient reported outcome (PRO) endpoints: patient global ratings of concept versus patient global ratings of change, a case study among osteoporosis patients
Source: Health Qual Life Outcomes. 2016 Feb 19;14:25. doi: 10.1186/s12955-016-0427-5 (PMC4759933; doi:10.1186/s12955-016-0427-5)
Supplement: Additional file 2: Figure S2. — ROC curves identifying the best cut point (BCP, indicated by the arrow) of OPAQ-PF change scores for an improvement of 1 point on Mobility, Physical Positions, and Transfers ratings of change and ratings of concept at weeks 2 (no recent fracture patients) and 12 (recent fracture patients). (DOCX 188 kb) [file 12955_2016_427_MOESM2_ESM.docx]

*Additional file 2: Figure S2: ROC curves identifying the best cut point (BCP, indicated by the arrow) of OPAQ-PF change scores for an improvement of 1 point on Mobility, Physical Positions, and Transfers ratings of change and ratings of concept at weeks 2 (no recent fracture patients) and 12 (recent fracture patients)*

| **No Recent Fracture Patients** | **Recent Fracture Patients** | **No Recent Fracture Patients** | **Recent Fracture Patients** |
| --- | --- | --- | --- |
| **Week 2** | **Week 12** | **Week 2** | **Week 12** |
| **Global Ratings of Change** | | **Global Ratings of Concept** | |
| ***Mobility*** | | | |
| 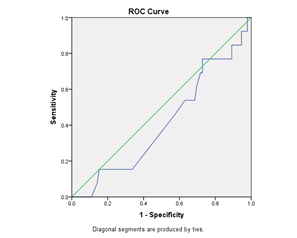 | 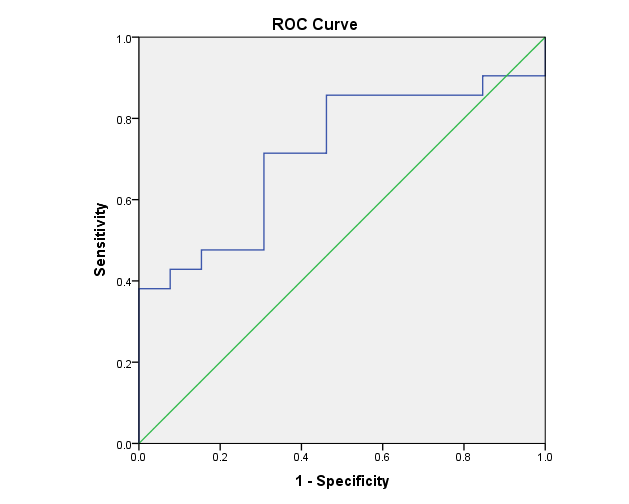 | 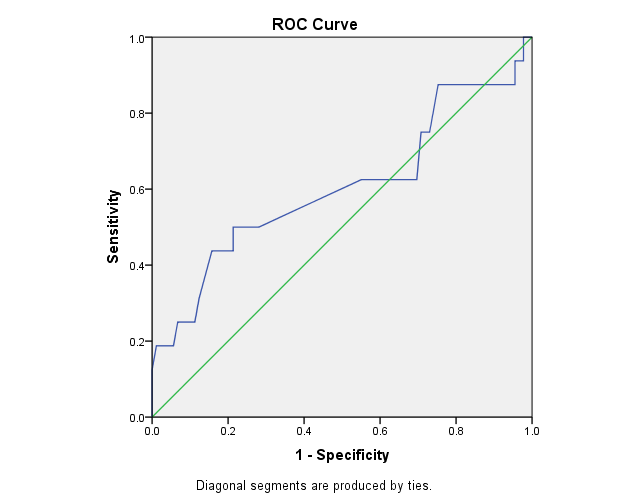 | 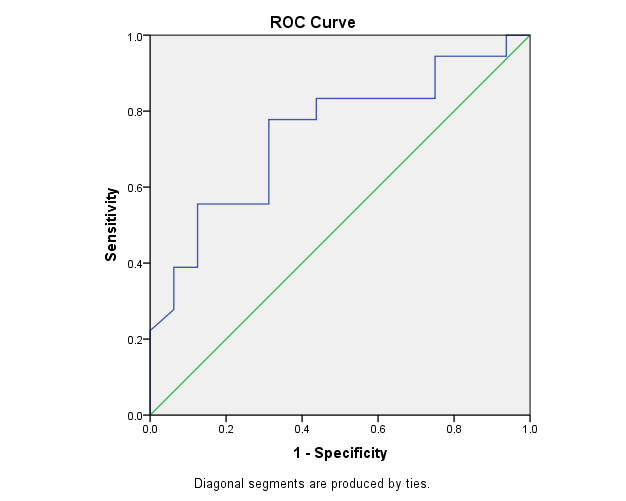 |
| ***Physical Positions*** | | | |
| 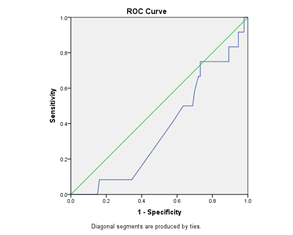 | 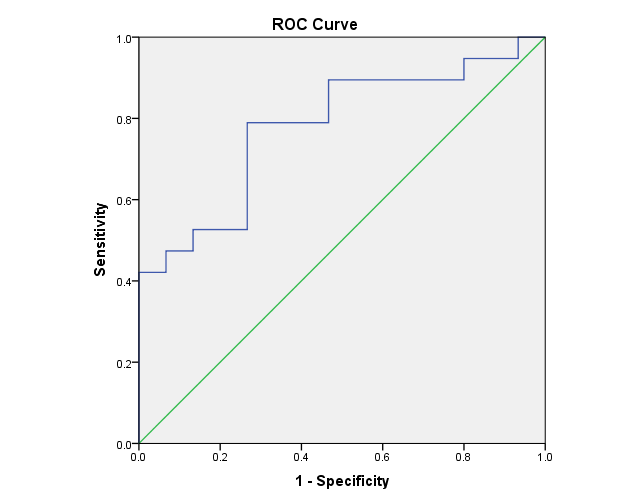 | 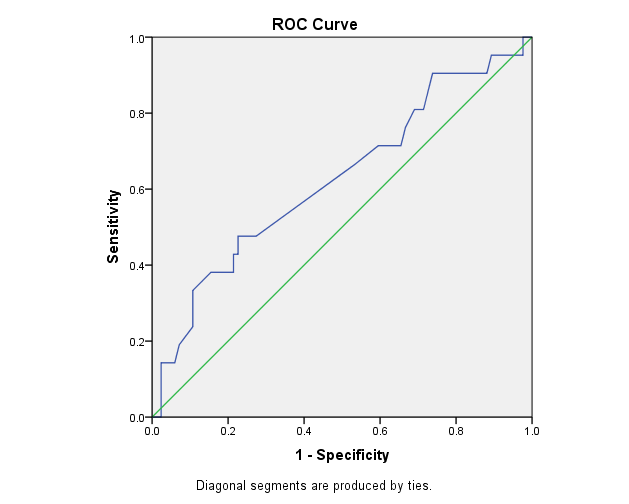 | 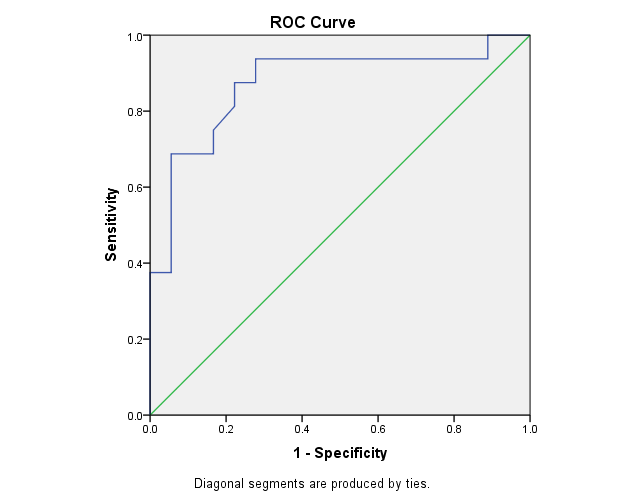 |
| ***Transfers*** | | | |
| 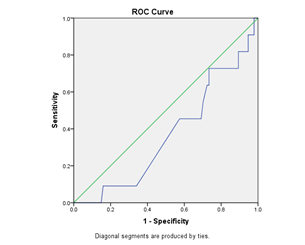 | 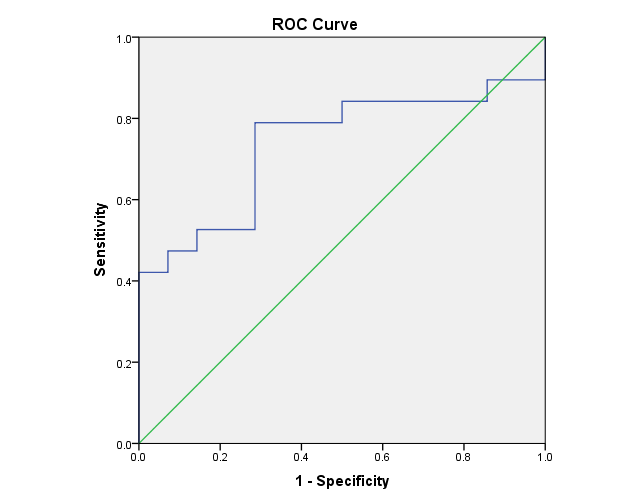 | 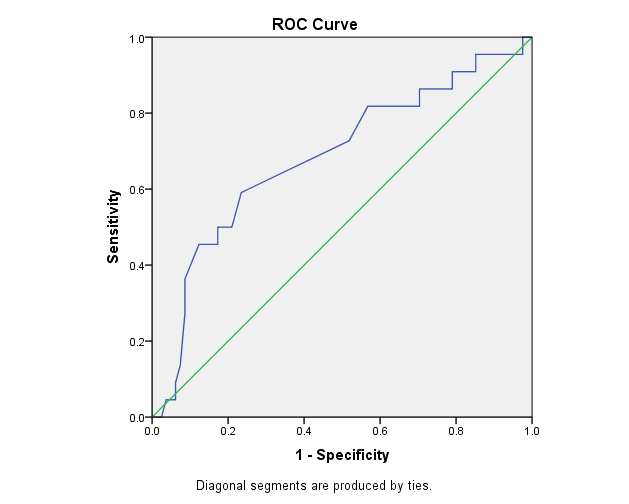 | 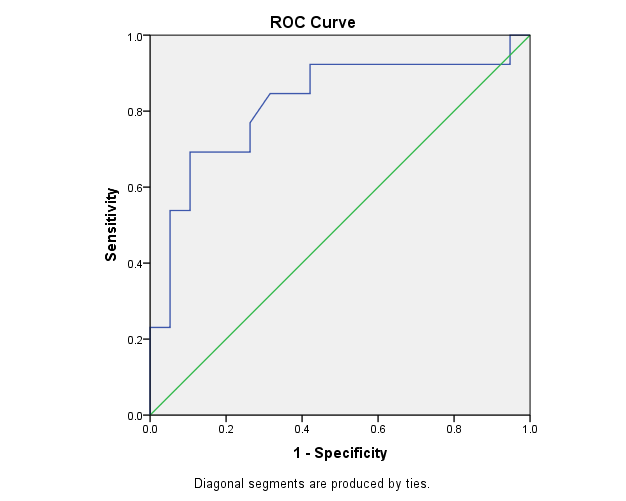 |
